# A community-based service enhancement model of training and employing Ear Health Facilitators to address the crisis in ear and hearing health of Aboriginal children in the Northern Territory, the Hearing for Learning Initiative (the HfLI): study protocol for a stepped-wedge cluster randomised trial

**DOI:** 10.1186/s13063-021-05215-7

**Published:** 2021-06-16

**Authors:** Kelvin Kong, Alan Cass, Amanda Jane Leach, Peter Stanley Morris, Amy Kimber, Jiunn-Yih Su, Victor Maduabuchi Oguoma

**Affiliations:** 1 University of Newcastle, John Hunter Children’s Hospital, Newcastle, NSW 2300 Australia; 2 Menzies School of Health Research, Charles Darwin University, 58 Rocklands Drive, Darwin, NT 0810 Australia; 3 Royal Darwin Hospital, Darwin, NT Australia; 4 Health Research Institute, University of Canberra, Canberra, ACT Australia

**Keywords:** Stepped-wedge cluster randomised trial, Health Facilitator, Otitis media, Aboriginal and Torres Strait Islander

## Abstract

**Background:**

Almost all Aboriginal children in remote communities have persistent bilateral otitis media affecting hearing and learning throughout early childhood and school years, with consequences for social and educational outcomes, and later employment opportunities. Current primary health care and specialist services do not have the resources to meet the complex needs of these children.

**Method/design:**

This stepped-wedge cluster randomised trial will allocate 18 communities to one of five 6-monthly intervention start dates. Stratification will be by region and population size. The intervention (Hearing for Learning Initiative, HfLI) consists of six 20-h weeks of training (delivered over 3 months) that includes Certificate II in Aboriginal Primary Health Care (3 modules) and competencies in ear and hearing data collection (otoscopy, tympanometry and hearScreen), plus 3 weeks of assisted integration into the health service, then part-time employment as Ear Health Facilitators to the end of the trial. Unblinding will occur 6 months prior to each allocated start date, to allow Community Reference Groups to be involved in co-design of the HfLI implementation in their community. Relevant health service data will be extracted 6-monthly from all 18 communities. The primary outcome is the difference in proportion of children (0 to 16 years of age) who have at least one ear assessment (diagnosis) documented in their medical record within each 6-month period, compared to control periods (no HfLI). Secondary outcomes include data on sustainability, adherence to evidence-based clinical guidelines for otitis media, including follow-up and specialist referrals, and school attendance. Structured interviews with staff working in health and education services, Ear Health Trainees, Ear Health Facilitators and families will assess process outcomes and the HfLI broader impact.

**Discussion:**

The impact of training and employment of Ear Health Facilitators on service enhancement will inform the health, education and employment sectors about effectiveness of skills and job creation that empowers community members to contribute to addressing issues of local importance, in this instance ear and hearing health of children.

**Trial registration:**

ClinicalTrials.gov NCT03916029. Registered on 16 April 2019.

**Supplementary Information:**

The online version contains supplementary material available at 10.1186/s13063-021-05215-7.

## Administrative Information (SPIRIT checklist)

1. Title

A community-based service-enhancement model of training and employing Ear Health Facilitators to address the crisis in ear and hearing health of Aboriginal children in the Northern Territory, the Hearing for Learning Initiative (HfLI): study protocol for a stepped-wedge cluster randomised trial.

2. Trial registration and status

ClinicalTrials.gov (NCT03916029) registered 16th April 2019.

Protocol version number (HREC-approved: v4 on 5th April 2019, v14 on 8th Sep 2020)

Recruitment began 3rd & 18th Feb 2020 (Intervention Training began in two pilot communities)

Recruitment began 1st and 6th October 2020 (Intervention Training began in first two randomised communities)

Recruitment completed estimated 31st Dec 2023

3. Protocol version

Document name: Hearing for Learning Initiative - SWCRT protocol - v7 - Trials journal.

Last saved: 1-Apr-21 at 04:01.

Created on: 19/05/2020 12:56 PM.

4. Funding

The Hearing for Learning Initiative is funded by the Australian Commonwealth Government, Northern Territory Government, and The Balnaves Foundation. These funding partners have no role in the design of the study, collection, analysis, or interpretation of data or in writing the manuscript.

5. Roles and Responsibilities

5a. Contributorship

Protocol contributors are Associate Professor Kelvin Kong, University of Newcastle (concept, funding, draft revisions and read final version)

Professor Alan Cass, Menzies School of Health Research, Charles Darwin University (concept, funding, draft revisions and read final version)

Professor Amanda Jane Leach, Menzies School of Health Research, Charles Darwin University (concept, design, funding, wrote the first draft, draft revisions and read final version)

Professor Peter Stanley Morris, Menzies School of Health Research, Charles Darwin University and Royal Darwin hospital (concept, design, funding, draft revisions and read final version)

Dr Jiunn-Yih Su, Menzies School of Health Research, Charles Darwin University (draft revisions and read final version)

Dr Victor Maduabuchi Oguoma, Menzies School of Health Research, Charles Darwin University (biostatistician expertise draft revisions and read final version)

5b. Sponsor contact information

The trial Sponsor is the Menzies School of Health Research, PO Box 41096 Casuarina, Northern Territory 0811. Contact details: p +61 8 8946 8600. www.menzies.edu.au.

5c. The study sponsor and funders had no role or authority over study design, and will not have any role during collection, management, analysis, and interpretation of data; writing of the report; or the decision to submit the report for publication.

5d. Committees

The coordinating centre is at Menzies School of Health Research. Staff consist of the Hearing for Learning Initiative Program Manager, Data Manager and Analyst, Administrative Assistant, Community Liaison Officers, Senior Clinical Training Research Officer, and Clinical Training Research Officers. The role of the clinical trial team is to plan, monitor and manage the HfLI, undertake community consultation, manage stakeholder engagement including establishing and managing the HfLI governance structure, manage the HfLI finances and reporting requirements, deliver the HfLI training and employment (intervention), and collect and analyse data.

The governance structure of the Hearing for Learning Initiative consists of an Advisory Board, Community Reference Groups, Integration Working Group, Training Working Group, independent Data Safety Monitoring Board, and Research Executive Committee. It includes quorum rules to ensure Aboriginal and Torres Strait Islander leadership. Members’ roles are to provide advice that will ensure the trial is consistent with best-practice, is successfully completed, maintains trial integrity, is culturally appropriate and safe for Aboriginal and Torres Strait Islander communities, and has a high likelihood of successful transfer to policy and practice if the outcome is positive.

## Introduction

### Background and rationale

#### Research questions

Among Aboriginal children (0 to 16 years of age) living in the Northern Territory, does training and employment of local Ear Health Facilitators (the HfLI), compared to no HfLI improve surveillance, detection and evidence-based management of otitis media and hearing loss, during 6-monthly intervals? Among Aboriginal communities in the Northern Territory, is on-country training, integration and employment of Ear Health Facilitators, compared to no HfLI, an effective workforce enhancement model?

#### Prevalence of otitis media in the Northern Territory

Otitis media (OM or middle ear inflammation) is a complex condition with a continuum of diagnostic categories including otitis media with middle ear effusion (OME or ‘glue ear’), acute otitis media without perforation (AOMwoP or bulging ear drum), acute otitis media with (small) perforation (AOMwiP or recent ‘runny ears’), or chronic suppurative otitis media (CSOM or long term ‘runny ears’ with a larger perforation), dry perforation (DP or inactive CSOM), or tympanostomy tube otorrhoea (TTO, ear discharge through a TT). All forms of OM cause some level of hearing loss. Disease and hearing loss is considered more significant for the child if OM is bilateral (both ears). From our surveillance of vaccine impact on OM prevalence between 2001 and 2013 across almost 30 remote NT communities, the overall prevalence of any form of OM in young children has been consistently at or above 70% although CSOM prevalence has declined from around 24% to around 15% [1, 2]. Past and current longitudinal birth cohort studies confirm early age of onset and persistent OM and conductive hearing loss throughout the first 3 years of life [3–5, 6]. Recent analyses using administrative data linkage reveal the ongoing impact of early childhood chronic otitis media and conductive hearing loss throughout life course, on early childhood development, school attendance, child maltreatment and involvement with the youth justice system [7–10].

#### Primary health care workforce

The Northern Territory Healthy Under 5 Kids is a comprehensive program of scheduled child health assessments for children living in remote communities. An evaluation reported that ear examinations were completed in less than 40% of scheduled visits, that no hearing screens were done and that tympanometry was rarely used (< 5%) while a diagnosis was made in 10% [11]. Although essential specialist services are available, they are delivered by fly-in/fly-out (FIFO) clinicians and consequently waiting lists for audiology and ENT consultations are in the thousands and wait-times are years [12]. Failure to meet child health program schedules and evidence-based practice in ear and hearing health [11, 12] is linked to high turnover of the health workforce [13–15], inadequate clinical skills, lack of diagnostic equipment and poor knowledge of ear and hearing health needs of children. These suggest an inability of the current workforce of Primary Health Care to provide the required care for the ear and hearing health in this setting.

In addition, an evaluation reported in 2014 found that uptake of the 2010 Recommendations for Clinical Care Guidelines on the Management of Otitis Media in Aboriginal and Torres Strait Islander Populations (the 2010 OM Guidelines) has been low [16]. The evaluation therefore recommended that in the future, consideration should be given to having two levels of training, i.e. one for those who diagnose and medically manage children with OM and another less complex for those who do opportunistic screening. The HfLI has been proposed and developed in order to address the health service workforce shortfall and to respond to the second recommendation. Training community-based Ear Health Facilitators will focus on ear and hearing surveillance and facilitating delivery of evidence-based clinical care recommendations according to the 2020 OM Guidelines [17].

Design of the HfLI has been informed by a systematic review of models for Indigenous primary health care service delivery, [18] a qualitative assessment of barriers and enablers to child health care service delivery [19], and impact of CQI on quality of care for Aboriginal and Torres Strait Islander children [20]. The systematic review found 62 studies meeting inclusion criteria to answer the question “what are characteristics of successful [Indigenous] PHC service delivery” [21]. Eight characteristics were identified; access, community participation, CQI, culturally appropriate and skilled workforce, culture, flexible approach to care, holistic, self-determination and empowerment. Strategies for embedding and respecting culture were identified. The qualitative assessment identified primary drivers as staff capability to deliver high-quality care, availability and use of clinical information systems and decision support tools, embedding of quality improvement processes and data-driven decision-making, appropriate and effective recruitment and retention of staff, and community capacity, engagement and mobilisation for health [19]. Suggested strategies included mechanisms for increasing clinical supervision and support, staff retention, reorientation of service delivery, use of information systems and community health literacy. Data from 59 Australian primary health care centres demonstrated that participation in annual CQI improved quality of care for Aboriginal and Torres Strait Islander children, particularly hearing assessments [20].

The HfLI aims to improve uptake of guidelines. We found evidence from a meta-analysis of data from 23 studies and almost 1400 practices that Practice Facilitators in Primary Health Care settings can increase adoption of evidence-based guidelines by almost 3-fold [22]. Furthermore, and importantly also for our research question, there is specific evidence that the diagnostic accuracy of a health care facilitator with no formal ENT training and using video-otoscopy is superior or similar to that of a general practitioner using traditional otoscopy [23]. Such innovation in Primary Health Care services could make substantial differences to delivery of ear and hearing health care but requires rigorous assessment in the unique setting of the NT.

Very few Indigenous health programs are rigorously evaluated. A recent systematic review identified 118 papers describing evaluations of 109 interventions [18]. Most (36) were before/after comparisons, only 9 used an experimental design of which 3 were cluster-randomised controlled trials. One third used mixed qualitative and quantitative data, and most reported process outcomes only (rather than health outcomes). Another systematic review of the role of Aboriginal Health Workers (AHWs) and Liaison Officers in Australian acute care settings found just 5 studies with consistent identification of the importance of the AHW role in patient communication and continuity of care [24]. A further systematic review examined the factors influencing accountability (social, political, provider and organisational) relationships of AHWs in the Australian health system [25]. Barriers to AHWs working to their full capacity stemmed from the clash between their value as cultural brokers with organisational and provider accountability. Our trial will evaluate qualitative and quantitative process and health outcomes in terms of community engagement in co-design, delivery and appropriateness of training (Certificate II and ear and hearing screening technical skills), integration of Ear Heath Facilitators into service delivery, impact of additional jobs and employment, impact on ear and hearing surveillance and adherence to evidence-based clinical practice guidelines. Clinical outcomes will be reported as secondary quantitative outcomes, although power to detect statistically significant differences at the population level (e.g. proportion of children with improved ear and hearing health) and within the study time frame will be limited.

A stepped-wedge cluster randomised trial (SW-CRT) design was chosen with unit of randomisation being the community health service. Compared to the simple parallel arm implementation, the SW-CRT allows for staggered introduction of the HfLI to all participating communities. Concurrent delivery of the HfLI to ten communities was not feasible nor was the concept of controls popular. At conclusion of our trial, 20 communities will have experience of the HfLI and be poised for uptake of the program, should it be successful and sustainable, by the Government and Aboriginal Community Controlled Health sector. Expansion across NT remote communities is anticipated, with potential for application to other health priorities.

We acknowledge the difficulties of the SW-CRT design, including having to adjust for calendar time, as systematically more assessments are accrued later under intervention condition than under control conditions. It is also possible that the effect of the Ear Health Facilitator may vary over the duration of the study. The open cohort allows for continuous entry, and as the only participant inclusion criteria is age 0 to 16 years, the risk of selection bias is low. Adjustment for repeat measures (6-monthly surveillance and follow-up ear assessments) has been accounted for in sample size and analysis plan.

Our trial incorporates a co-design approach to implementation, led by Community Reference Groups, and a strengths-based approach that respects the cultural, linguistic and relationship knowledge of community members.

The comparator (control periods) represents standard care. To maintain interest and communication with communities during the control period, we will offer annual 1-day workshops for resident health service staff in the prevention, diagnosis and management of OM. Such brief workshops have always been available to services and will not contaminate our outcome measure. We also anticipate little impact of within cluster contamination.

## Objectives

The aim of the HfLI is to improve the ear and hearing health of Aboriginal children living in the Northern Territory.

Our primary hypothesis is that implementation of the HfLI model of health service facilitation will increase ear and hearing health surveillance (documented in the medical records) among children 0 to 16 years of age. Our secondary hypotheses are that compared to control periods; the HfLI will improve evidence-based management of OM and hearing loss, improve access to specialist services, improve follow-up care, reduce the prevalence and severity of OM and hearing loss and improve family and children’s quality of life, school attendance and performance. We also hypothesise that the co-design and delivery of training and employment on country has benefits for the Ear Health Trainees, the Ear Health Facilitators, families and professional health and education staff, including the opportunities this model might provide for training and employment in other health areas, and other service sectors of importance to their community.

### Trial design

**Fig. 1:**
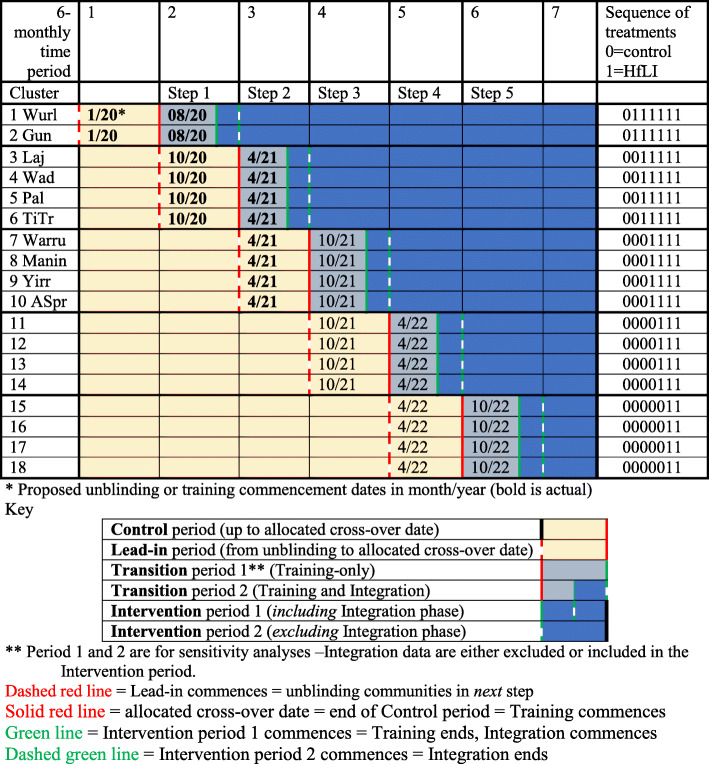
Design matrix (actual dates inserted prospectively) with dates (month/year) proposed for unblinding and training commencement

The HfLI is designed as an open cohort stepped-wedge cluster randomised superiority trial. Eighteen communities will be allocated according to a computer-generated randomisation list stratified by community size and location, to one of five (steps) 6-monthly HfLI start dates (Fig. 1, Tables 1 and 2).

**Table 1:** Definitions applied to the Hearing for Learning Initiative SW-CRT (see Fig. 1)

Cluster (unit of randomisation)	18 Northern Territory Aboriginal communities (clusters) allocated 2:4:4:4:4 to 5 start dates (sequences). Stratified by NT region (Top End or Central) and size (large or small)
Cluster period (a grouping of observations by time of measurement and cluster)	7 including baseline (i.e. 1 baseline, 5 training or transitions, and 6 Initiatives). Each cluster period has a duration of 6 months
Number of sequences	5 sequences. The order of sequence is 0111111, 0011111, 0001111, 0000111 and 0000011 (0 = control and 1 = initiative)
Number of clusters randomised to each sequence	Variable (2 in 1 sequence and 4 in 4 sequences)
Number of time periods	7 including baseline
Duration of time between each step	6 months
Transition period	3-month training period

**Table 2:** Participant timeline (actual dates to be inserted prospectively)

Community numbers	Unblind	Lead-in phase (within control periods)	Cross-over dates (transition periods)	Integration and employment commences (intervention periods)
1–2	Month 1 TP 1	Months 1 to 10* TP1-2	Months 10 to 12 TP2	Month 1 TP3
3–6	Month 10* TP2	Months 10 TP2 to month 4 TP3	Months 4 to 6 TP3	Month 7 TP4
7–10	Month 4 TP3	Months 4 to 10 TP4	Months 10 to 12 TP4	Month 1 TP5
11–14	Month 10 TP4	Month 10 TP4 to month 4 TP5	Months 4 to 6 TP5	Month 7 TP6
15–18	Month 4 TP5	Months 4 to 10 TP6	Months 10 to 12 TP6	Month 1 TP7

*Anticipated delay due to COVID-19 ** TP: 6-monthly TimePeriod (Fig 1)

### Methods: participants, interventions and outcomes

#### Study setting

The Northern Territory (NT) of Australia, which covers approximately 1.3 million km^2^, is sparsely settled, with a population of less than 250,000 people, 30% of whom identify as Aboriginal Australians. Most (81%) Aboriginal Territorians live in remote communities. Analysis of resident health workforce in 53 remote primary care clinics found a median of 2.0 nurses, 0.6 Aboriginal health practitioners (AHPs), 2.2 other employees and 0.4 additional agency-employed nurses. Workforce turnover is very high, with half the nurses and AHPs leaving a community within 4 months [13]. Professional support for ear and hearing health is provided by tele-otology and scheduled intermittent visits by audiologists and surgical outreach teams.

#### Eligibility criteria

Communities (Table 3) were eligible for randomisation if the HfLI was approved by a governing body (Aboriginal Cooperation or Local Authority) on or before 19 February 2020, if the population includes at least 100 children (0 to 16 years of age), the local health service prioritises improved ear and hearing health services, there is a local school, there are appropriate facilities for the training to be delivered, there is a Community Reference Group or interest to form such a group to ensure cultural safety and appropriateness and the HfLI has permission from the relevant Land Council to work in the community. Communities with a moratorium on research or with significant unrest or feasibility barriers were excluded.

**Table 3:** Participating community information (from NT Government websites)

Name	R	S	Pop	Pop < 14yo	Data	Health	School	Audiol booth	Local government councils	Land council
Ali Curung	C	S	580	142	PCIS	CAHS	DoE	Yes	Barkly	CLC
Alice Springs (CAAC)	C	L		> 300	Comm	CAAC	DoE/CE	Yes	Alice Springs	CLC
Ampilatwatja	C	S	491	140	Comm	AHCAC	DoE	Yes	Barkly	CLC
Galiwin’ku	T	L	2453	670	Comm	Miwatj	DoE	Yes	East Arnhem	NLC
Gunbalanya	T	S	1312	246	PCIS	TEHS	DoE	Yes	West Arnhem	NLC
Kalkaringi	T	S	392	103	Comm	KWHB	DoE	No	Victoria Daly	CLC
Katherine (Wurli)	T	L		> 300	Comm	Wurli	DoE/CE	Yes	Katherine	NLC
Lajamanu	T	S	703	215	Comm	KWHB	DoE	Yes	Central Desert	CLC
Maningrida	T	L	2712	617	PCIS	TEHS	DoE	Yes	West Arnhem	NLC
Milikapiti	T	S	471	101	PCIS	TEHS	DoE	No	Tiwi Islands	TLC
Milingimbi	T	L	1439	335	Comm	Miwatj	DoE	Yes	East Arnhem	NLC
Minjilang	T	S	290	81*	PCIS	TEHS	DoE	No	West Arnhem	NLC
Nauiyu	T	S	444	124	PCIS	TEHS	CE	No	Victoria Daly	NLC
Palumpa	T	S	458	144	PCIS	TEHS	DoE	No	West Daly	NLC
Pirlangimpi	T	S	436	93*	PCIS	TEHS	DoE	No	Tiwi Islands	TLC
Ti Tree	C	S	317	80*	PCIS	CAHS	DoE	Yes	Central Desert	CLC
Wadeye	T	L	2679	724	PCIS	TEHS	CE	Yes	West Daly	NLC
Warruwi	T	S	457	130	PCIS	TEHS	DoE	No	West Arnhem	NLC
Wurrumiyanga	T	L	1837	392	PCIS	TEHS	CE	Yes	Tiwi Islands	TLC
Yirrkala	T	S	951	240	Comm	Miwatj	DoE	No	East Arnhem	NLC

R, region; C, Central Australia; T, Top End. S, size; S, small; L, large (L and S for stratification purposes only ; ≥ or < 300 children < 16 years of age). Pop, population size published by NTG for 2016; Pop< 14yo, population Aboriginal children less than 14 years of age published by NTG for 2016. For Alice Springs and Katherine, the numbers are imprecise but > 300. *Population less than 14 years old is ≤ 100 but estimated to be > 100 for 0 to 16 years of age. Data: PCIS, Patient Care Information System. Comm, Communicare. Health: CAHS, Central Australian Health Service. TEHS, Top End Health Service. CAAC, Central Australian Aboriginal Congress. AHCAC, Ampilatwatja Health Care Aboriginal Corporation. Miwatj: Miwatj Health Aboriginal Corporation. KWHB: Katherine West Health Board. Wurli: Wurli-Wurlinjang Aboriginal Health Service. School: DoE, Department of Education. CE, Catholic Education. Audiol booth: Sound proof audiology booth in community. Land Council: CLC, Central Land Council. NLC, Northern Land Council. TLC, TiwiLand Council

Children will be eligible for ear assessments if they are 0 to 16 years of age and their parents have provided written informed consent. No child will be excluded.

Ear Health Trainees will be eligible for training if they are recommended by the Community Reference Group, Elders, the health service, school or childcare centre, have a Working with Children (OCHRE) card, pass a Literacy and Numeracy assessment and are interested in addressing the ear and hearing problems in their community.

Ear Health Facilitators will be those Ear Health Trainees who pass the three Cert II modules in Aboriginal Primary Health Care and who pass ear and hearing clinical competency assessments, who are recommended by the Community Reference Group, who want to apply for employment by the health service and are successful.

##### Eligibility criteria for participation in process evaluation

The impact of the HfLI will also be assessed via annual structured questionnaires or interviews. Participants will be eligible for structured questionnaires or interviews on the basis of their role in the service or community. All Ear Health Trainees and Ear Health Facilitators will be eligible. All Community Reference Group members will be eligible if they have attended meetings during both the lead-in period and HfLI period. Health service staff will be eligible if they have been resident staff during both the control and HfLI periods. This will include the clinic manager, doctor, nurse, child health nurse and Aboriginal health practitioner. Other non-resident visiting health service staff and those providing specialist ear and hearing-related services on a fly-in/fly-out basis will be eligible if they have provided services during both the control and HfLI periods. The school principal and inclusion teacher will be eligible. School teachers, teacher aids and school attendance officers will be eligible if they were in the community during both the control and HfLI periods; one pre-school, one primary school, one senior school, one teacher aid and one attendance officer will be selected at random. Parents of children 0 to 16 years of age will be eligible, and older children if parental consent is provided. Parents will be selected at random, stratified by whether they have a child with or without OM or hearing loss identified by the Ear Health Facilitator. Where a participant selected at random refuses consent, another participant will be selected.

### Interventions

Control period will be from commencement of the trial (date of unblinding first step, January 2020) to each community allocated cross-over date (Fig. 1, Table 2). The comparator consists of standard care with brief in-service video workshops for health professionals if requested (Table 4).

**Table 4:** Description of intervention and usual care groups according to the Template for Intervention Description and Replication (TIDieR)

Item	TIDieR criterion	Intervention (initiative)	Usual care
1	Brief name	Hearing for Learning Initiative (HfLI): training and employment of Ear Health Facilitators. The intervention is multi-modal—training, integration, employment and service delivery to achieve the primary outcome of increase ear and hearing service enhancement	Usual Primary Health Care (PHC), plus the HfLI will provide annual half-day in-service clinical workshops during the control period if required.
2	Why: describe any rationale, theory or goal of the elements essential to the intervention	The NT experiences ongoing high prevalence of otitis media, hearing loss and associated disadvantage among Aboriginal children This may be explained in-part by: • Poor adherence to evidence-based guidelines in PHC • Long wait lists for specialist services and high rates of non-attendance • High turnover of remote health workforce There is evidence that • Health facilitators can improve guideline adherence • Video otoscopy by non-health professionals is as good as GPs The HfLI applies a multi-modal approach to addressing the burden of disease through expanding PHC services with local Ear Health Facilitators trained in use of new technologies for ear and hearing assessments.	Control period half-day workshops will keep control communities interested and included. Control period half-day workshops will be evaluated and are not expected to contaminate the control periods.
3	What (materials): describe any physical or informational materials used in the intervention, including those provided to participants or used in intervention delivery or in training of intervention providers	Materials for training Ear Health Facilitators (EHFs) Cert II modules in Aboriginal Primary Health Care (Workplace Health and Safety, Working with Aboriginal clients, write simple workplace information) (HLT20113), delivered under a Third-Party Agreement with the Registered Training Organisation, Central Australian Remote Health Development Services, Alice Springs Ear and Hearing Health Training manual (Menzies) 2020 OM Guidelines. Otitis media guidelines for Aboriginal and Torres Strait Islander children. OMapp, posters etc. Ear and hearing surveillance equipment consist of otoscope, tympanometer, hearScreen, Parent-Evaluated Listening and Understanding Measure (PLUM) and Hear & Talk Scale (HATS). [Hearing Australia].	Control period half-day workshops will not provide materials. PHCs have standard materials including Central Australian Rural Practitioners Association, (CARPA) manual, Guidelines for Otitis Media in Aboriginal and Torres Strait Islander populations (2020).
4	What (procedures): describe each of the procedures, activities or processes used in the intervention, including any enabling or support activities	We established support for the intervention through a Participation Agreement signed at Departmental level and including a Schedule of Services for each participating community which sets out the roles and responsibilities of each collaborating partner, as well as for the Ear Health Facilitator when working with the health service, community, or school. Training consists of 120 h delivered on-country during 6 weeks over a 3-month period. The first 2 weeks cover the Cert II modules, followed by 4 weeks of skills training in ear and hearing health screening including otoscopy, video otoscopy, tympanometry, and hearScreen. Integration consists of three 2-day visits by the Senior CTRO who will work with the Ear Health Facilitator and the health service to establish induction, supervision, workplans, work station and secure storage of data. Ongoing training will include advanced data management skills, obtaining informed consent and working with specialist services (audiology and tele-otology). Employment of Ear Health Facilitators in the health service (0.5FTE) will be reimbursed by the HfLI. The service will provide supervision and resources to enable day-to-day case load and priorities to be achieved. Refresher training: 6-monthly on-site visits by the CTROs will assess EHF retention	Control period half-day workshops will include theory, epidemiology, microbiology and briefing on best practice based on CARPA and 2020 OM Guidelines. Community health services provide induction for remote area nurses, doctors and Aboriginal health practitioners (AHPs) including a 1h seminar on ear and hearing health, often given in part by Menzies Ear Health Research Program staff. Training in video otoscopy and tympanometry is not generally provided.
5	Who provided: for each category of intervention provider (for example, psychologist, nursing assistant), describe their expertise, background and any specific training given	Trainers: registered nurses who have Cert IV in Training and Assessment are employed as Clinical Training Research Officers (CTROs). Trainees receive in-community Cert II modules in Aboriginal Primary Health Care and training in ear and hearing health, video otoscopy, tympanometry and hearScreen. Ear Health Facilitators: Ear Health Trainees are community members nominated by the Community Reference Group, health service, school and other community members. Eligibility includes live in community, speak language of the community and English, pass literacy learning numeracy, have clearance for working with children (OCHRE card) and police check. They should have an interest in the ear and hearing health of children in their community. Ear Health Trainees are paid a casual wage during training. To graduate as EHFs, the trainees must not have missed more than 2 days of training, must pass the three Cert II modules in Aboriginal Primary Health Care and have demonstrated knowledge and clinical competency in ear and hearing screening. The EHFs are selected for employment by the health service, with recommendations from the training team and Community Reference Group. The EHFs then undergo an Integration phase, delivered by a trainer together with health service staff to ensure safe and effective placement into the workforce. The Health Service is reimbursed the wages of the EHF by the HfLI until the end of the project. Service delivery: ear and hearing screening will be delivered by EHFs with supervision from a health professional.	The HfLI trainers will provide annual half-day in-service clinical workshops during the control period if required. Control period half-day workshops will be via questionnaires.
6	How: describe the modes of delivery (such as face to face or by some other mechanism, such as internet or telephone) of the intervention and whether it was provided individually or in a group	Training: delivered by two trainers or trainer and a community engagement officer face to face in community for a group of trainees. Appropriate venues are selected by the Community Reference Group. These should meet WHS standards, have power, bathroom facilities (wash basin) and space for 5 trainees and two trainers. Integration of the EHF within the Health Service: delivered face to face with trainer over 3 visits, plus phone support. Health service champion and supervisor to manage WHS, day to day case load. Service delivery: EHFs deliver ear and hearing screening services in community face-to-face for children 0 to 16 years of age.	Workforce: Control period half-day workshops will be evaluated via questionnaires. Service delivery: Usual care face to face ear assessments for individual children who present to the health service. School screening.
7	Where: describe the types of location where the intervention occurred, including any necessary infrastructure or relevant features	20 remote or rural communities across the NT were eligible if there were at least 100 children 0 to 16 years of age and there was a PHC Service that prioritises ear and hearing health, a school and infrastructure to support training. PHC services will be provided with a video otoscope, tympanometer and hearScreen device/s for use by the EHFs. Service delivery: EHFs deliver ear and hearing services in community at home, in school, kindergarten or at the clinic. The Schedule of Services sets out the responsibilities of each partner including infrastructure requirements.	Control period half-day workshops will be evaluated via questionnaires.
8	When and how much: describe the number of times the intervention was delivered and over what period of time, including the number of sessions, their schedule, and their duration, intensity or dose	Training intervention: 120 h delivered on-country during 6 weeks over a 3-month period commencing at date allocated. EHF integration into Health Service: over 3 months during 3 visits by trainer in addition to phone support. Service delivery: Ear and Hearing services intervention: the EHFs (on average 0.5 FTE per community) will screen at least 100 children every 6 months in each community. The EHFs will also assist families and teachers with strategies to enhance school attendance and learning.	Control period half-day workshops will be evaluated via questionnaires.
9	Tailoring: if the intervention was planned to be personalised, titrated or adapted, then describe what, why, when and how	Training: up to five Ear Health Trainees will be paid on a casual basis to undertake the training course. Trainees must not miss more than 2 days of the 24-day course to qualify for employment as an EHF. The trainers who fly-in fly-out are not able to make up for missed training sessions for individuals. The group can negotiate most suitable dates for training to occur, within constraints of the program, flights, accommodation and commitments to other communities. Employment: the fulltime equivalent (FTE) for employment of Ear Health Facilitators will be 0.5 per community (10 FTEs in total). For statistical analyses, the target sample size for each community is 100 children seen per 6 months. Service delivery: priority children will be determined by the service and according to 2020 OM Guideline recommendations.	N/A
10	Modifications: if the intervention was modified during the course of the study, describe the changes (what, why, when, how)	Not at this stage	N/A
11	How well (planned): if intervention adherence or fidelity was assessed, describe how and by whom; if any strategies were used to maintain or improve fidelity, describe them	All aspects of adherence will be measured. Training: attendance at training will be documented via timesheets. Fidelity will be assessed by Cert II processes and clinical competency by the trainers. Employment: strategies used to maintain or improve fidelity. The EHFs will be supported by their supervisor and nominated champion and mentor and will have regular contact via phone with their trainers. Evaluation questionnaires and interviews will be used to monitor adherence and fidelity. Service delivery: trainers will visit communities 6-monthly to deliver refresher training and identify fidelity (technical skills and data collection). Six-monthly data retrieval from the PCIS or Communicare systems will show the productivity of the intervention in terms of services delivered by the initiative.	Control period half-day workshops will be evaluated via questionnaires.
12	How well (actual): if intervention adherence or fidelity was assessed, describe the extent to which the intervention was delivered as planned	This will be reported in terms of process and impact for workforce and health measures.	Control period half-day workshops will be evaluated via questionnaires.

The intervention comprises three phases of training, integration and employment of local community members as Ear Health Facilitators. Senior staff in each health service and school will be asked to note the Schedule of Services in the HfLI Participation Agreement, which sets out the roles and responsibilities of each collaborating partner, as well as for the Ear Health Facilitator when working with the service or school.

#### Training phase

Details are provided in Table 4. Briefly, training consists of on-site accredited and non-accredited training (120 h in total) delivered by registered health professionals with Certificate IV qualifications in training and assessment, advanced skills in ear and hearing health screening checks and knowledge of the 2020 Otitis Media Guidelines for Aboriginal and Torres Strait Islander children [17] and the CARPA manual [26].

#### Integration phase

Ear Health Facilitators who are selected for employment will transition to a health service integration phase, supported by the HfLI training team (Table 4).

#### Employment phase

Service delivery (Table 4). Briefly, the Ear Health Facilitator(s) will obtain informed consent for ear and hearing surveillance of at least 100 children every 6 months. Otoscopy, tympanometry and hearScreen findings will be entered into the child’s electronic health record (PCIS or Communicare). A diagnosis and care plan will be made in consultation with the child’s doctor, nurse or Aboriginal health practitioner. The Ear Health Facilitator will assist with implementation of the care plan with the family. The Ear Health Facilitator will support families and children to improve school attendance and support teachers who have children in their class who need ear or hearing assistance.

Ear Health Facilitators who resign or lose their job will not be replaced. If there is more than one Ear Health Facilitator employed in the community (i.e. job sharing), the remaining employee will be offered a higher full time equivalent. Alternatively, the health service may employ an Ear Health Trainee who successfully completed the Cert II and clinical competency training; however, the service must deliver the integration phase according to the HfLI protocols. The health service will be reimbursed by the HfLI for employment of the Ear Health Facilitator(s) until the end of the project.

Regular 6-monthly 1-day refresher training workshops for Ear Health Facilitators will be a part of the HfLI program during the employment phase. Sustainability will be measured.

We do not anticipate discontinuing or modifying the HfLI. An independent Data Safety and Monitoring Board (iDSMB) will review progress of the trial. The terms of reference will include stopping rules regarding safety concerns. The HfLI will cease in a community if the Community Reference Group, the Local Authority and the community health and education services request this in writing (i.e. participant withdrawal). Communities will not be replaced.

Sustainability of the workforce is a key goal and retention of Ear Health Trainees and Ear Health Facilitators critical to the HfL Initiative concept. Strategies to improve adherence include casual payment for Ear Health Trainees and payment of Ear Health Facilitators salaries. Adherence to HfLI protocols (attendance, integration phase, refresher training, health and education services) will be evaluated by the training team (via time sheets), HfLI champions (via interviews) and the regular review of aggregated administrative data (ear and hearing screening and diagnosis, follow-up and school attendance).

There will be no restrictions on concomitant activities in participating communities including concomitant employment and training, clinical services such as audiology, speech therapy, surgeries and all other relevant care and education assistance.

### Outcomes

#### Primary outcome—surveillance enhancement

For the resident population of 0- to 16-year-old children, we will report the proportion who have at least one ear *observation,* and the proportion who have at least one ear *diagnosis* documented in their medical records within each 6-month period.

### Outcome definitions

#### Resident population

The resident population has a (primary) medical record with the participating community health service.

#### Ear observation

An ear observation is defined as data recorded using otoscopy or video otoscopy with tympanometry or pneumatic video otoscopy. Ear observation data therefore includes visual observations of the tympanic membrane (e.g. colour, position, perforation size, and mobility if using pneumatic otoscopy) and tests of mobility (tympanogram data such as peak height).

#### Hearing observation

A hearing observation is defined as data recorded using parent reported information (scored using structured questionnaires; Parent-Evaluated Listening and Understanding Measure, PLUM or Hear & Talk Scale, HATS) and hearScreen data (pass, fail) [Hearing Australia].

#### Presumptive diagnosis

Presumptive diagnosis is a diagnosis derived from analysis the ear or hearing observation data.

#### Clinical diagnosis

Clinical diagnosis is a diagnosis derived by a health professional from the ear and hearing observation data or by direct clinical assessment.

#### Management plan

A management plan is defined as the actions recommended as a result of a clinical diagnosis or hearing loss assessment.

Ear Health Facilitators will conduct ear and hearing observations. These observations can be made at venues outside the clinic and where appropriate for the family and Ear Health Facilitator. The data will be recorded on standardised forms on tablets or paper, and then entered into the child’s medical record. The Ear Health Facilitator will document whether the child’s ear and hearing observations are normal (no further action) or not normal (refer to the clinic doctor, nurse or AHP for a diagnosis). The health professional will be responsible making the diagnosis and management plan and entering these into the child’s medical record. Appointments for follow-up and specialist referrals will be assisted by the Ear Health Facilitator if required.

#### Secondary outcomes—ear and hearing observations and presumptive diagnoses (in the last 6 months)

For the resident population of 0- to 16-year-old children, we will report the proportion who have had the following: an ear observation attempted; otoscopy, tympanometry or both; a presumptive diagnosis; a clinical diagnosis; and a documented management plan.

For the resident population of 0- to 5-year-old children, we will report the proportion completing parental questionnaires. For the resident population of 5- to 16-year-old children, we will report the proportion with the following: hearScreen tests completed, presumptive hearing loss documented, an appropriate documented management plan and an appropriate documented referral for each specialist service.

#### Secondary outcomes—training progress

For those community members that completed an expression of interest, we will report the proportion who are eligible, enrolled, pass Certificate II in Aboriginal Primary Health Care, meet clinical competency criteria (by child age group), complete integration, are employed as Ear Health Facilitator(s) and the total FTE employed per 200 children.

#### Secondary outcomes—Ear Health Trainee assessment of training course and trainer performance

Qualitative measures: Ear Health Trainees will be asked to provide weekly feedback on each component of the training course (weeks 1 and 2: Cert II modules, weeks 3 to 6: ear and hearing observation training) for each of the following domains: what they enjoyed, most useful things learned for their future role as an Ear Health Facilitator, things that were difficult to understand or learn, valuable knowledge and skills learned, how the HfLI training program can be improved and other comments.

#### Secondary outcomes—trainer assessment of Ear Health Trainee technical skills

The trainers will assess evidence that the Ear Health Trainee can follow procedures and protocols to conduct otoscopy, tympanometry, and hearScreen can communicate effectively with children and caregivers using a client-centred approach, can maintain confidentiality, can maintain infection control principles and can assist in undertaking a health promotion activity in their community.

#### Secondary outcomes—Ear Health Trainee self-assessment

The Ear Health Trainees will make self-assessment scores (5-step ranking) pre-training and post-training in measures of confidence (five questions), knowledge (five questions) and clinical skills (6 questions). We will report the change in self-assessment scores.

### Secondary outcomes—integration and employment

Integration measures will be Ear Health Facilitator workplace attendance, punctuality, data collection and data entry skills (Redcap, PCIS or Communicare), attendance at supervisor meetings, case load and attendance during outreach service visits (audiologist and surgical outreach).

Subgroup analyses will be by child age group (0 to 3, 4 to 5, over 5 years of age) and by the five Priority groups recommended by the 2020 OM Guidelines.

Sensitivity analyses will compare data from control and HfLI intervention periods with or without the integration period.

Participant timeline (Table 2 and Fig. 1)

- Control periods: For all communities, the control period begins in year one at unblinding of the first allocated cross-over date (January 2020). For each community, the control period ends at commencement of their training and includes the lead-in phase.
- Lead-in phase: For each community, the period between unblinding of their allocated cross-over date to commencement of training (3 to 6 months)
- Transition period: The period between commencement and completion of training (3 months).
- Integration phase: The period between completion of training and end of integration into the health service (3 months).
- Intervention period: The period between completion of training (or end of Integration phase in sensitivity analysis) and end of TimePeriod 7.

#### Sample size

Sample size for the SW-CRT is 18 communities, each having at least 100 children 0 to 16 years of age. The sample size calculation is based on the proportion of participants who receive an ear assessment during the study period. The method of sample size calculation for a cohort SW-CRT design [27] was adapted using numerical simulation. Current evidence suggests that about 33% of children under 5 years of age received a completed ear assessment between 2011 and 2016 in the Northern Territory [11]. We would assume an absolute increase of 10% in the proportion of children who receive an ear assessment to be feasible and clinically important. Given a control proportion of 0.329 and an absolute difference of 0.1 using a two-sided test at 5% level of significance, our sample size of average 90 children per cluster (community) per 6 month period will achieve a total sample size of 1620 at 98% power assuming an intra-cluster correlation coefficient (ICC) of 0.06, a cluster auto-correlation coefficient (CAC) of 0.7 and individual autocorrelation (IAC) due to repeated measurement of children receiving ear diagnoses. We conducted sensitivity analysis using different effect sizes, power, ICC and CAC scenarios to determine if our required number of clusters will be adequate for our study. The result indicates that across a range of ICCs (0.02–1), CAC (0.6–0.8), baseline proportions (0.23–0.53) and effect sizes (0.10–0.20) our sample size of 18 clusters (communities) and 5 step lengths will achieve over 80% power to detect absolute increases between 10% and 20%. In the unlikely event of communities withdrawing, our sensitivity analysis shows that we will still have adequate power assuming we end up with 16 communities, allowing for withdrawal of two communities [28].

#### Recruitment

Strategies for recruiting 20 communities (includes two pilot communities) before commencement of the trial involved written applications (where required) and face-to-face consultations with the Aboriginal Medical Services Alliance of the Northern Territory (AMSANT), Aboriginal Community Controlled Health Organisations: Miwatj Aboriginal Corporation, Katherine West Health Board, Sunrise Health, Anyinginyi (Tennant Creek), Ampilatwatja Health Aboriginal Corporation, Central Australian Aboriginal Congress, Wurli Wurlinjang Health Service Board, Local Authorities for each community, Regional Councils (Barkly, Central Desert, East Arnhem, West Arnhem, Victoria Daly, West Daly, Katherine, Central Desert, Tiwi Islands, West Macdonnell) and Land Councils (Central Land Council, Northern Land Council, Tiwi Land Council), senior public servants in the health and education departments of the NT Government, the NT Top End and Central Australian health services, health service managers, primary healthcare managers, Remote Medical Practitioners, the NT Hearing Health Service Outreach Program audiologists and ENT surgical outreach team. NT Department of Education (including Families as First Teachers), Catholic Education NT, and school principals, teachers and/or inclusion support officers in the above communities have also been consulted. A community eligibility form was designed to collect data on feasibility of delivering the HfLI. Information was also obtained from websites. The NT Government’s Bushtel website was used to estimate the population of children 0 to 16 years, confirmed by the health services (Table 3).

### Methods: assignment of interventions (for controlled trials)

#### Allocation sequence generation

Communities will be randomly grouped into two stratification factors—region (Central vs. Top End) and size (small vs. large). These will be further assigned randomly into five different 6-monthly HfLI start dates with transition from control to HfLI being in sequences 0111111, 0011111, 0001111, 0000111 and 0000011 (0 = control and 1 = HfLI).

#### Allocation concealment mechanism

All communities were recruited prior to randomisation which was conducted at a single time point. Cross-over dates for each cluster will be concealed until the lead-in period, to reduce differential attrition. The statistician provided the allocation list (code/strata/start date) to the Menzies Senior Data Base Manager not involved in the HfLI delivery who maintains blinding until 3 to 6 months (lead-in phase) prior to each of the five HfLI start dates. Each group of communities will be unblinded by the independent Data Manager and provided to the Program Manager. The Program Manager will inform these communities and commence the lead-in phase (Table 2).

#### Implementation

The statistician not involved in the HfLI delivery will conduct the stratified allocation with clusters divided into distinct strata before random allocation within each stratum, using Stata 15.1 (StataCorp, College Station, TX).

#### Enrolment and consent (see Additional file 2)

Enrolment and consent of communities will be performed by the Program Manager and one of the investigators. This will involve the process described above. When 18 communities provide written expressions of interest in participation or other written approvals (i.e. consent), no further consultation will be undertaken. Communities that do not respond will be assumed to have refused consent and will be excluded (i.e. the first 18 will be enrolled). All community approvals will be provided to the ethics committees for confirmation of ethical approval.

#### Enrolment of child participants

For our primary outcome, the denominator is the number of resident children 0 to 16 years of age. Ethical approval for waiver of individual consent will be sought to retrieve relevant deidentified ear and hearing data from the medical records. A data access agreement will be executed with the relevant health services (Department of Health or ACCHOs) for this purpose.

For child participants receiving the HfLI (ear and hearing health checks by the Ear Health Facilitators), consent will be required. All age-eligible children whose parents consent to participation will be eligible for enrolment. Written informed consent will be obtained by the Ear Health Facilitators who will be trained by the Clinical Research Training Officers. To minimise selection bias, participant consent will be encouraged by multiple parties including Community Reference Group members, the school and health service staff and the Ear Health Facilitators. The open cohort design allows for continuous recruitment of children.

#### Enrolment of cluster level participants

For impact and program process evaluation questionnaires, written informed consent will be required from health and education service providers, Community Reference Group members, the Ear Health Trainees, Ear Health Facilitators and families. Consent will be obtained by members of the clinical trial team which includes the Ear Health Facilitators.

#### Blinding (masking)

Blinding will be maintained until 6 months prior to the training start date for each unblinded community (Fig. 1). Participant and research team blinding regarding the control-HfLI status cannot be achieved following assignment to a start date (Fig. 1). Administrative data from all communities will be retrieved 6-monthly for aggregated analyses. The Data Manager and Analyst will be aware of the cross-over dates and how many communities have transitioned from control periods to HfLI periods. However, the identity of communities will not be provided in the aggregated administrative dataset.

We do not anticipate the need for the Data Manager and Analyst to have any individual participant unblinded. The iDSMB will review process and progress of the trial implementation and data collection. Stopping rules will consider safety only. However, if there are major concerns about trial futility, such as withdrawal of more than four communities, poor engagement in training or other impacts such that an effect is unlikely to be detectable, or if the aggregated data suggests superiority of the HfLI, the iDSMB may request analysis of outcomes according to control and HfL Initiative periods. The Senior Data Managers at Menzies will hold the unblinded allocation of community start dates.

### Methods: data collection, management and analysis

#### Data collection methods

Data retrieval and analysis, trends analysis and retrospective data collection for all NT Government serviced communities included in the Primary Care Information System (PCIS) system database for the period of 2015–2019 will be used in calculating the pre-existing global trends in the targeted trial clinical outcome measures across remote NT communities prior to baseline. A similar process of trend analysis of data from communities using Communicare is planned.

#### Pilot phase

A pilot of the HfLI will be undertaken in two communities, one that uses the PCIS system and the other the Communicare system. Data fields relative to ear and hearing health and our targeted trial clinical outcome measures will be identified in each system for harmonisation. The pilot phase will test data collection and data entry methods (paper-based forms, tablets (Redcap) or direct database entry).

#### Trial phase

Data from all 18 participating communities (PCIS and Communicare) for the period from the start to the end of the trial will be retrieved 6-monthly using the data-identity separation principles for data retrieval and data linkage.

Ear assessment data entered and retrieved from the PCIS or Communicare systems by all health professionals and by Ear Health Facilitators will contribute to the analyses. Data collected by the Ear Health Facilitators alone (paper-based forms or tablets (REDCap) can be used to determine the contributions by the Ear Health Facilitators.

#### Communities as participants

Data will be collected from all 20 communities (including two self-nominated pilot communities) from baseline to end of the trial. Retention of communities prior to their start date, during the pre-HfLI period, will be encouraged through regular communications about the overall trial milestones and annual in-service 1-day workshops for resident health service professionals on evidence-based guidelines for OM in Aboriginal and Torres Strait Islander children. Retention of communities during the training, integration and HfLI periods will also be encouraged by the training team, the HfLI governance members including the Community Reference Groups, the study Champions, Ear Health Trainees and the Ear Health Facilitators. For communities who formally withdraw, we will negotiate the terms to ensure ongoing primary outcome data collection can occur. The trial has a participation agreement with key decision makers and services which will cover this.

#### Ear Health Facilitators as participants

Retention of the Ear Health Facilitators will be encouraged through a dedicated workplace integration plan, local mentors, local health and education professionals as HfLI champions, advice from the Community Reference Group and a help line to the Training team at Menzies. Employment of the Ear Health Facilitators by the health service will be funded by the HfLI until the end of the HfL Initiative. FTE will be determined by each community and budget allocation. Part-time and job share arrangements that suit the community cultural protocols will be a priority.

#### Children 0 to 16 years of age as participants

For our primary outcome, relevant ear and hearing data will be retrieved from the medical records of all children 0 to 16 years of age who reside in the 18 participating communities. Participation of families and their children in the ear and hearing checks will be encouraged. The HfLI aims to train and employ Ear Health Facilitators in community to ensure that families have confidence in the ear and hearing checks for their children. The Ear Health Facilitators will have state-of-art diagnostic equipment that will enable them to conduct these checks outside the clinic at places more convenient to families and children. Parental consent for the Ear Health Facilitators to conduct children’s ear and hearing checks will be required. The Community Reference Group and the Ear Health Facilitators will advise on best practice in informing parents about the HfLI, through ear health promotion activities at networking events across the community.

### Data management

#### Data entry

Each child’s ear and hearing health assessment made by the Ear Health Facilitators may be collected at non-clinic sites, such as the kindergarten, home or school. Standardised ear observation forms or tablets will be used when off-site. These forms will be held in locked storage at the health service. Data will be entered (asynchronously if collected off-site) into the child’s electronic medical record (PCIS or Communicare) either directly by the trained Ear Health Facilitator, by the trained Ear Health Facilitator under supervision or by the child’s health care provider who receives the ear observation data from the Ear Health Facilitator.

#### Database

For government clinics, standard PCIS ear and hearing-related fields will be used. For Communicare, a dedicated template has been designed for the project, including identical fields as the PCIS system, in addition to standard Communicare fields. The data fields on the paper forms, tablets, Communicare and PCIS systems will be harmonised. Data from questionnaires conducted verbally by the training or independently by participants will be collected on paper-based forms or tablet (into a purpose-built Redcap data entry form). Paper forms and tablets will be stored in secure offices at the health service. All paper-based forms will be scanned to a secure email address at Menzies School of Health Research. Original forms will be transported to Menzies by the training team or via post and using a tracking system for secure storage in locked filing cabinets. Data on tablets will be uploaded to a secure database at Menzies.

### Statistical methods

The intention to treat principle will be adhered to, where clusters will be analysed according to their time of switching between control and HfLI. Randomisation is conducted as soon as all communities have provided support of the HfL Initiative in their community and prior to the commencement in any of the participating communities. Unblinding of the communities allocated to the next 6-monthly cross-over date will be done 6 months prior to that cross-over date, to allow for a lead-in phase of community preparation that is still the control period. To allow for delays in implementation, a separate per protocol analysis will be performed with the assessments now placed into one of the three categories: control period pre-allocated cross-over date (including the lead-in period of 6 months), post-allocated cross-over date but pre-implementation (the training phase) and post-implementation (from commencement of employment and including integration phase). The distribution of results across the control group will be compared with the HfLI group

Characteristics of individuals and clusters in the HfLI will be summarised by their exposure status [29]. Demographic characteristics of children will be summarised as proportions and/or means (SD) or medians (IQR) by control and HfLI group. For a sub-set of the population, risk factor questionnaires with parents will be used to examine the distribution of known confounders (such as birth weight, smoke exposure from pre-conception, breast feeding, household crowding, etc.) between control and HfLI periods.

#### Primary outcome

The primary outcome is the proportion of children who receive an ear assessment (diagnosis) within 6 months. A multilevel mixed-effects generalised linear model will be used. This will involve fitting a binomial regression model with fixed effect for HfLI status and time as a categorical variable and a random intercept for cluster and coefficient for time to account for both within—and between—period intra-cluster correlations [27]. Estimates of treatment effect will be presented as odds ratios and the 95% confidence interval using a logit link function.

#### Secondary outcomes

##### Proportions

Secondary outcomes on binary scale include the proportion of children (1) with OM or hearing loss who receive a case management plan, (2) with a diagnosis requiring follow-up who receive an ear assessment within 10 days, (3) who receive screening otoscopy, (4) who receive screening tympanometry or pneumatic otoscopy, (5) 5 to 16 years of age who receive hearScreen, and (6) < 5 years of who receive parent listening and understanding measure and hearing and talking score (PLUM and HATS) assessments. For these outcomes, the approach applied to the primary outcome will be employed—estimates of treatment effect will be presented as odds ratios and 95%CI following a multilevel mixed-effects generalised linear regression model.

##### Rates

Episodes of AOM, OME, AOMwiP, CSOM or DP over the trial period will be summarised as counts according to treatment groups. The effect of the HfLI will be determined using multilevel mixed-effects negative binomial regression expressed as incidence rate ratio (IRR) and the 95% CI. Time will be fitted as a categorical fixed effect and random coefficient and random intercept for cluster. The same approach will be followed to estimate the treatment effect on rate of appropriate use of antibiotics for (1) persistent otitis media with effusion (pOME), (2) AOM without perforation, (3) AOM with perforation, (4) CSOM (topical) in the last 6 months, (5) rate of appropriate specialist referral (audiology), and (6) rate of appropriate specialist referral (ENT).

##### Subgroup analyses

There may be possibilities of differences in characteristics of populations across the 18 communities during the study period that may confound with the effect of the HfLI. In subgroup analysis, we will adjust for age, sex and factors associated with socio-economic disadvantage in Indigenous children. The subgroup analysis will be carried out as either a stratified analysis or inclusion of an interaction term between each subgroup variable, HfLI factor and time. In all analysis, statistical significance will be set at 5% significance level (*p* < 0.05). Analysis will be conducted using Stata 15.1 (StataCorp LLC, College Station, TX, USA).

The analysis population will be children 0 to 16 years of age who are resident in the participating community. The proportion of the population that receive services will be reported. There will be no imputation for those not seen. We will report participant flow (clusters and participants) for control and HfLI periods, the number of clusters and participants who were assessed for eligibility, were randomly assigned, received intended treatment, and were analysed for the primary outcome.

### Methods: monitoring

#### Data monitoring

The primary responsibilities of the iDSMB are (i) to periodically review and evaluate accumulated study data for participant safety, study conduct and progress and, where appropriate, efficacy and (ii) to make recommendations to the clinical trial investigators and the Indigenous Reference Group of the Menzies Child Health Division concerning the modification, continuation and/or termination of the trial. Detailed Terms of Reference for the iDSMB have been drafted, including items to be reviewed, recommendations for modification, potential stopping rules, roles of iDSMB members and investigators. Whenever blinded data are presented to the iDSMB, the code for unblinding will be made accessible in the case of need for immediate unblinding of the study. It is essential that the confidentiality of Interim Reports is maintained so as not to change the investigators’ outlook and participation, based on results that are not complete.

#### Harms

We expect a very low rate of clinical adverse events. Ear screening (otoscopy, tympanometry or hearScreen™ tests) can be uncomfortable or unpleasant but very rarely cause harm. No baseline data are available for adverse events of ear assessments or hearScreen™ tests, as these are not captured in routine data collection by primary health care services, audiologists or ENT surgeons. Solicited adverse events will include persistent bleeding following an ear observation.

Unintended effects (pros and cons) of the HfLI will be sought via questionnaires and interviews from participants including parents, children, Ear Health Trainees, Ear Health Facilitators, Community Reference Group members and health and education service staff.

#### Auditing

No audit of trial conduct is planned. Contact details of the ethics committees will be available to all participants should they have concerns about trial conduct.

### Ethics and dissemination

#### Research ethics approval

The Hearing for Learning Initiative (HfLI) trial protocol was approved by the Human Research Ethics Committee of the Northern Territory Department of Health and Menzies School of Health Research (NHMRC Reg no. EC00153), conditional approval on 3 December 2018 and full approval on 3 April 2019. The Central Australian Human Research Ethics Committee (Ref CA-19-3308) provided full approval on 5 April 2019.

#### Protocol amendments

Protocol amendments will be reported to the ethics committees in accordance with guidelines. Important amendments are not anticipated beyond the pilot phase in two communities; however, should these occur, they will be reported to stakeholders via our governance groups including the Community Reference Groups and on the trial registry (ClinicalTrials.gov).

#### Consent or assent

The training team will provide relevant information to participants and obtain their consent to conduct interview questionnaires for trial evaluation. The Ear Health Facilitators will be trained in providing participant information and in obtaining informed consent from parents (and children) to perform ear and hearing screening, enter data into their child’s medical record and the Menzies database and collect information via structured interviews about their child’s risk factors for OM. A waiver of individual consent has been be sought for data linkage at the end of the study. No additional use of participant data nor specimen collection is planned.

#### Confidentiality

Personal information collected from child participants and their families will be restricted to risk factors for OM. These data will be managed as per the ear and hearing screening data, on paper-based forms or tablets with procedures for secure storage and transfer to a central password-protected server at Menzies.

#### Declarations of interest

All investigators declare no financial or competing interests for the overall trial or at any participating site.

#### Access to data

Menzies Senior Data Manager is the trial data custodian. The HfLI Statistician, Data Manager and Analyst, Program Manager and trial investigators will have access to the final trial data set.

Funding partners will not have access to data.

#### Ancillary and post-trial care

The trial has clinical trial insurance to compensate participants who suffer harm from trial participation.

#### Dissemination policy

Trial results will be available after data collection from all 18 randomised communities has been completed and analysed, possibly towards late 2023. The results will be reported in aggregate to all communities in the form of clear summaries in plain English, along with recommendations, implications for policy and practice and future directions. Sensitivity analyses according to stratification (Top End and Central, community size large or small) will also be reported. Communities may request information specific to their community, but identification will not be revealed in the aggregate data. Authorship eligibility guidelines set out by the ICMJE will be followed. There are no plans to make public access to the full protocol, participant-level data or statistical code.

### Appendices

Model consent form and other related documentation given to participants and authorised surrogates can be provided if required.

### Supplementary Information

**Additional file 1.** Sample size simulation with 18 communities.

**Additional file 2. **Community Participation Information Letter.

**Additional file 3. **SPIRIT checklist.

## Data Availability

Menzies is the trial data custodian, managed by the Senior Data Manager. The HfLI Statistician, Data Manager and Analyst, Program Manager and trial investigators will have access to the final trial data set. Funding partners will not have access to data.

## Appendix

“How to administer HATS” available at: https://www.youtube.com/watch?time_continue=4&v=QchdHcSgU5A&feature=emb_logo

or 1:17 video at https://www.hearhappy.nal.gov.au/training-resources and 1:40 video “How to score the HATS and what the scores mean”.

“How to administer the PLUM” available at:

https://www.youtube.com/watch?time_continue=2&v=DkOVC-W9Epw&feature=emb_logo

or 1:00 video at https://www.hearhappy.nal.gov.au/training-resources and 1:23 video “How to score the PLUM and what the scores mean”

## Abbreviations

HfLI: Hearing for Learning Initiative
NT: Northern Territory
OM: Otitis media
EHF: Ear Health Facilitator
SWCRT: Stepped-wedge cluster randomised trial
AOM: Acute otitis media
OME: Otitis media with effusion
AOMwiP: Acute otitis media with perforation
CSOM: Chronic suppurative otitis media
DP: Dry perforation
ENT: Ear, nose and throat
PLUM: Parent Listening Understanding Measure
HATS: Hearing And Talking Score
ICC: Intra-cluster correlation coefficient
CAC: Cluster auto-correlation coefficient
IAC: Individual autocorrelation
