# Supplementary material for: A community-based service enhancement model of training and employing Ear Health Facilitators to address the crisis in ear and hearing health of Aboriginal children in the Northern Territory, the Hearing for Learning Initiative (the HfLI): study protocol for a stepped-wedge cluster randomised trial
Source: Trials. 2021 Jun 16;22:403. doi: 10.1186/s13063-021-05215-7 (PMC8207498; doi:10.1186/s13063-021-05215-7)
Supplement: Supplementary file 1 — Additional file 1. Sample size simulation with 18 communities. [file 13063_2021_5215_MOESM1_ESM.docx]

### APPENDIX TWO: Sample size simulation with 18 communities

Estimated power

Two-sided test

+-----------------------------------------------------------------------------------------+

| alpha power N pa pb diff iac cac icc tnclus nstep |

|-----------------------------------------------------------------------------------------|

| .05 .982 90 .329 .429 .1 .7 .7 .06 18 5 |

+-----------------------------------------------------------------------------------------+

Estimated power

Two-sided test

+-----------------------------------------------------------------------------------------+

| alpha power N pa pb diff iac cac icc tnclus nstep |

|-----------------------------------------------------------------------------------------|

| .05 1 90 .329 .429 .1 0 .6 .02 18 5 |

| .05 .8088 90 .329 .429 .1 0 .6 1 18 5 |

| .05 1 90 .329 .429 .1 0 .8 .02 18 5 |

| .05 .8097 90 .329 .429 .1 0 .8 1 18 5 |

| .05 .994 90 .329 .429 .1 .6 .6 .02 18 5 |

| .05 .793 90 .329 .429 .1 .6 .6 1 18 5 |

| .05 1 90 .329 .429 .1 .6 .8 .02 18 5 |

| .05 .7823 90 .329 .429 .1 .6 .8 1 18 5 |

| .05 .995 90 .329 .429 .1 .8 .6 .02 18 5 |

| .05 .8148 90 .329 .429 .1 .8 .6 1 18 5 |

| .05 .997 90 .329 .429 .1 .8 .8 .02 18 5 |

| .05 .8193 90 .329 .429 .1 .8 .8 1 18 5 |

+-----------------------------------------------------------------------------------------+
