# Supplementary material for: A community-based service enhancement model of training and employing Ear Health Facilitators to address the crisis in ear and hearing health of Aboriginal children in the Northern Territory, the Hearing for Learning Initiative (the HfLI): study protocol for a stepped-wedge cluster randomised trial
Source: Trials. 2021 Jun 16;22:403. doi: 10.1186/s13063-021-05215-7 (PMC8207498; doi:10.1186/s13063-021-05215-7)
Supplement: Supplementary file 2 — Additional file 2. Community Participation Information Letter. [file 13063_2021_5215_MOESM2_ESM.doc]

DATE

Professor Amanda Leach

Head, Ear Heath Research Program,

Child Health Division,

Menzies School of Health Research,

Darwin, NT. 0811

Dear Amanda,

**Re: Hearing for Learning Initiative**

Thank you for your application and for meeting with NAME OF ORGANISATION on DATE to request support for the *Hearing for Learning Initiative.*

I understand that the proposed initiative aims to work with communities in urban, regional and rural areas to establish reliable, sustainable, culturally appropriate integrated services that ensure that *every ear of every child is healthy and hearing every day and* will comprise:

1. **Establishment of a national coalition of Indigenous leaders across sectors and jurisdictions** to ensure the program meets the needs of Indigenous children in the NT and also across Australia who have ear disease, hearing loss and are at risk of not meeting their full potential in learning and achieving their goals.
2. **Implement and Evaluate the *Hearing for Learning Initiative* across the Northern Territory.** The *Hearing for Learning Initiative* will include training, employment and resourcing of up to 40 Indigenous community members as Ear and Hearing Health Clinical and Education Support Officers in 20 pilot sites across urban, rural and remote areas of NT. The Ear and Hearing Health Clinical and Education Support Officers will receive workforce preparation and clinical training onsite, supported regularly by Indigenous mentors, clinicians and teachers, and provided with key diagnostic equipment to support their work, including: tympanometers (plus maintenance for calibration), laptops and video otoscopes, and voroscopes (pneumatic). The Ear and Hearing Clinical and Education Support officers will also be trained to work in pre-schools and schools to ensure that the children with ear or hearing problems attend school, are assisted in listening or wearing their hearing aids, and that teachers are using techniques to improve learning of hearing impaired children, including using sound field and acoustic improvements.

I understand that the HfLI is interested to engage NAME OF ORGANISATION as a partner in this innovative, community-based, service enhancement model to address the ear crisis in ear and hearing health of Aboriginal children in the Northern Territory.

I understand that would include training, employing and mentoring local Aboriginal and Torres Strait Islander people as Ear and Hearing Health Clinical and Education Support Officers.

On behalf of the NAME OF ORGANISATION, I am pleased to provide this letter of support to Menzies School of Health Research. We look forward to partnering your team working on this initiative.

In practical terms, NAME OF ORGANISATION confirms support and engagement in the following ways:

- Non-professional community members being employed and trained to support health and education professionals by conducting ear and hearing tests and implementing case management plans.
- Integration of Ear and Hearing Clinical and Education Support Officers into current services to ensure sustainability of the program.
- Participating in stakeholder feedback on all levels of evaluation

I congratulate on your new initiative. The NAME OF ORGANISATION is looking forward to working with your team on this important project.

Yours Sincerely,

POSITION

NAME OF ORGANISATION.
